# Case report: A case report and literature review of complete trisomy 9

**DOI:** 10.3389/fgene.2023.1241245

**Published:** 2023-08-31

**Authors:** Chenxia Xu, Miaoyuan Li, Jianming Peng, Yanfang Zhang, Haijun Li, Guobing Zheng, Degang Wang

**Affiliations:** ^1^ Prenatal Diagnosis Center, Boai Hospital of Zhongshan, Zhongshan, Guangdong, China; ^2^ Department of Urology, Zhongshan People’s Hospital, Zhongshan, Guangdong, China; ^3^ The First School of Clinical Medicine,Jinan University, Guangzhou, Guangdong, China; ^4^ The Second School of Clinical Medicine, Southern Medical University, Guangzhou, Guangdong, China

**Keywords:** aneuploid, complete trisomy 9, chromosomal disorder, prenatal diagnosis, genetic counseling

## Abstract

Complete trisomy 9 is a rare and lethal chromosomal anomaly characterized by multisystem dysmorphism and central nervous system (CNS) malformations. This study presents a case of complete trisomy 9 with an unusual phenotypic association and investigates the genetic pathways involved in this chromosomal abnormality. Trisomy 9 leads to a wide range of organ abnormalities, and this research contributes to a better understanding of the phenotype associated with this rare aneuploidy. The literature on the phenotypes of fetuses with various systems affected by complete trisomy 9 was reviewed and summarized. Correct diagnosis and appropriate counseling based on the characteristics of previous reports of fetuses with trisomy 9 is essential in maternity care and clinical management. To provide guidance and help for clinical diagnosis, this study aimed to explore the clinical and genetic characteristics of trisomy 9 syndrome to improve clinicians’ understanding of the disease.

## Introduction

The prenatal diagnosis of complete trisomy 9 in fetuses presents several challenges. First, it is a rare condition during pregnancy, which may be unfamiliar to many doctors. Second, a significant number of affected fetuses do not survive *in utero*. Consequently, complete trisomy 9 is rarely observed and may not be initially considered or included in the list of potential diagnoses. Nonetheless, the accurate identification of fetuses with complete trisomy 9 remains crucial due to its poor prognosis. Chromosome 9 comprises approximately 141 million DNA base pairs, representing approximately 4.5% of the total DNA in cells. Chromosome 9 contains 2,466 genes, including 605 OMIM genes, and 162 of these 605 OMIM genes have been proven to be associated with diseases (data from https://www.gena.tech/; https://ghr.nlm.nih.gov/condition). The first report of trisomy 9 syndrome was published by Feingold and Atkins in 1973 (Feingold and Atkins, 1973). Complete trisomy 9 is characterized by the presence of an additional whole chromosome 9 in all cells, without evidence of mosaicism (Ferreres et al., 2008). Trisomy 9 is a fatal chromosomal disease, which mostly results in spontaneous abortion in early pregnancy. Trisomy 9 syndrome is a rare condition that affects multiple organ systems, including craniofacial dysmorphisms, cardiac abnormalities, genitourinary malformations, skeletal anomalies, and central nervous system abnormalities. Understanding the characteristics and manifestations of complete trisomy 9 can help doctors with early diagnosis. This knowledge can guide doctors in providing early interventions to minimize physical and psychological harm to pregnant women. For pregnant women, early detection and diagnosis of complete trisomy 9 can provide vital information to make early decisions, including further prenatal testing or the choice of pregnancy termination.

We present a case illustrating the complete form of this trisomy and performed a thorough review of the available literature to provide a comprehensive understanding of this syndrome. The aim was to help with the identification of clinical features and the performance of laboratory tests, prenatal genetic diagnosis, and genetic counseling for trisomy 9. We present a review of 59 cases of trisomy 9 to better define the phenotype and to determine its characteristics. Fetuses with complete trisomy 9 have multiple anomalies that can be readily detected prenatally by ultrasound. These anomalies primarily involve the craniofacial, cardiovascular, musculoskeletal, and genitourinary systems. However, some findings may be subtle and easily missed during routine ultrasound examinations (Sepulveda et al., 2003). Therefore, it is important to know the clinical characteristics of the various systems affected by trisomy 9 syndrome.

## Case report

**FIGURE 1:**
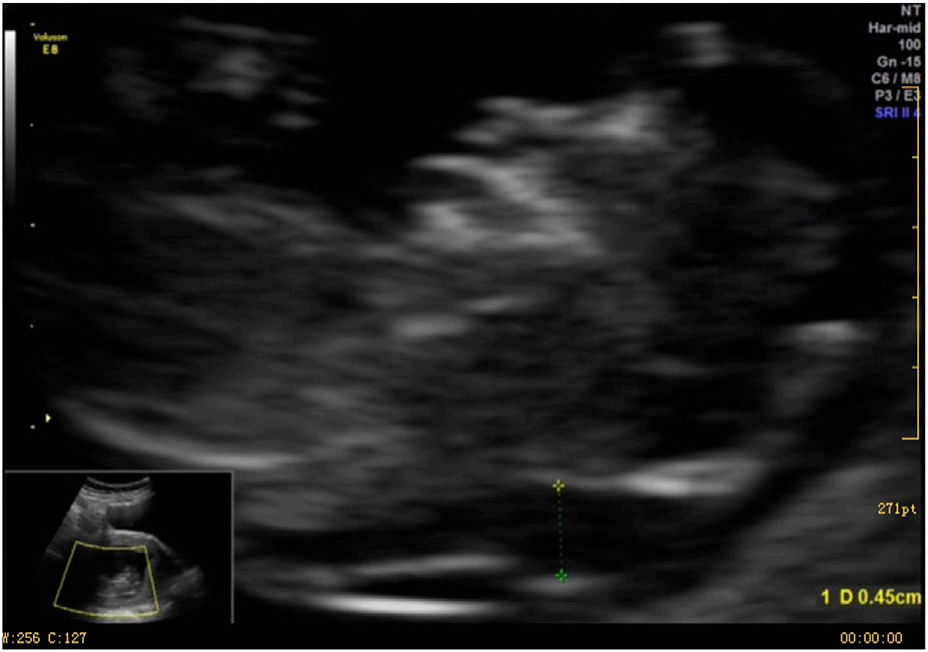
2D sonographic image of the fetus at 12^+2^ weeks showing NT = 4.5 mm (vertical section).

**FIGURE 2:**
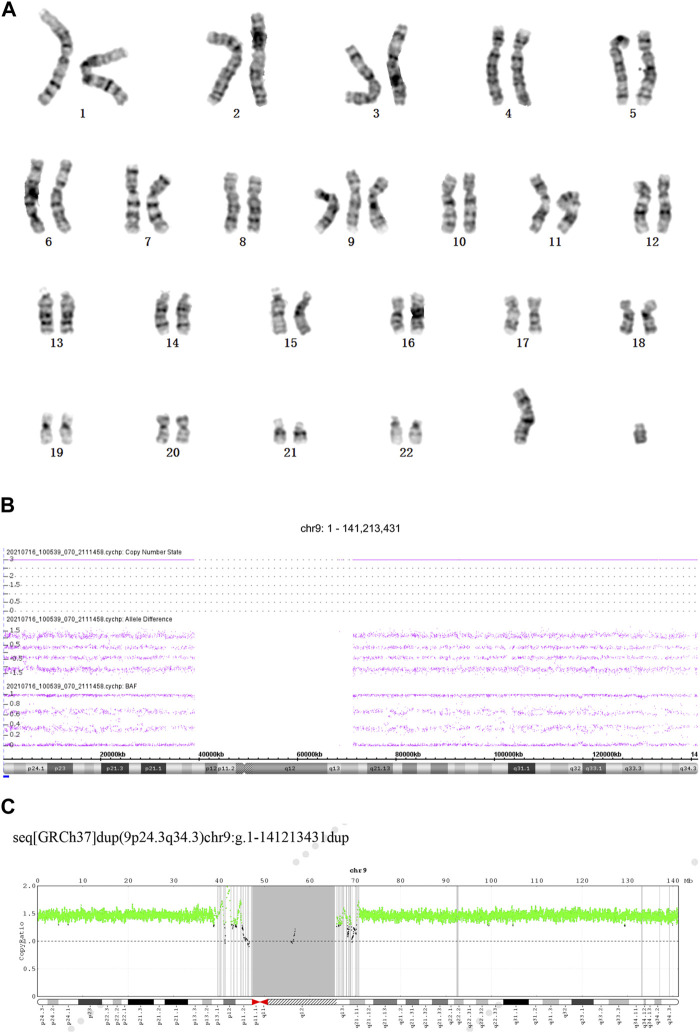
**(A)** Results of karyotype analysis showed 47,XY,+9. **(B)** Chromosomal microarray results of the amniocyte indicated trisomy 9. **(C)** CNV-seq assay results of the placenta, fetal skin tissue, umbilical cord blood, kidney, and heart indicated trisomy 9.

A 37-year-old female underwent routine fetal ultrasound examination at 12^+2^ weeks of pregnancy, which revealed thickened nuchal translucency. The examination was performed using a Voluson E8 ultrasound apparatus (GE Healthcare, Milwaukee, WI, United States) equipped with a multifrequency transabdominal RAB 4-8D probe. The procedures were performed according to the quality control standards of the British Fetal Medicine Foundation. As shown in Figure 1, thickened nuchal translucency (NT = 4.5 mm) was observed on fetal ultrasound. The woman had experienced a total of four pregnancies, including one ectopic pregnancy and one natural miscarriage at 8 weeks. She successfully delivered a healthy baby boy who is now 15 years old. The woman had no physical discomfort during the current pregnancy. Her partner was a 39-year-old healthy male. They had no history of medical conditions or medications, no abnormal family history, and no history of consanguineous marriages. At 19 weeks of gestation, fetal intrauterine growth restriction was noted on ultrasonography. The pregnant woman underwent amniocentesis at 19 weeks of pregnancy. Amniotic fluid specimens were collected by abdominal amniotic cavity puncture under the guidance of B-ultrasound. The results of G-banding karyotype analysis and CMA indicated trisomy 9 (Figures 2A, B). The pregnancy was terminated at 24 weeks of gestation, and a series of clinical examinations and genetic testing were conducted. The study protocols were approved by the Ethical Review Committee of the Boai Hospital of Zhongshan (KY-2023-004-47). A next-generation sequencing-based copy number variation (CNV-seq) assay was performed on the labor induction tissue. CNV-seq assay results of the placenta, fetal skin tissue, umbilical cord blood, kidney, and heart indicated trisomy 9 (Figure 2C). The examination revealed multiple anomalies (Supplementary Figure S1). The fetus’s face showed typical indications of trisomy 9. The face was dysmorphic, with a broad forehead, blepharophimosis, low-set malformed ears with small lobes, a prominent nose with a bulbous tip, and micrognathia. The fetus’s mouth was similar to a fish’s mouth, and the fetus had a broad neck, postural anomalies, broad thumbs, and clubfeet. Both renal malformations were connected and limited joint movement was observed.

## Discussion

Complete trisomy 9 is typically associated with spontaneous abortion. Individuals with trisomy 9 seem either to die very early in embryonic life or survive to be born at term, many of the latter showing mosaicism (Saura et al., 1995; Saneto et al., 1998; Chen et al., 2023a). The prenatal diagnosis of trisomy 9 presents challenges in genetic counseling due to the need to differentiate between pseudo-mosaicism, fetal-placental discrepancy, and true trisomy 9. Studies have demonstrated varying levels of trisomy 9 mosaicism ranging from 99% to normal in different tissues (Tang et al., 2019; Ma et al., 2023). Trisomy 9 mosaicism tends to show different levels of mosaicism in various tissues. Low-level mosaic trisomy 9 at amniocentesis can be associated with a favorable fetal outcome (Chen et al., 2023b). When trisomy 9 mosaicism is suspected, genetic testing using uncultured cells is necessary to reflect the proportion of trisomy 9 mosaicism more accurately. Previous studies have suggested that the clinical phenotype of trisomy 9 mosaicism is similar to that of complete trisomy 9, while the clinical symptoms of trisomy 9 mosaicism are milder than those of complete trisomy 9 (Arnold et al., 1995; Saneto et al., 1998; Li et al., 2021). Individuals with low-level mosaic trisomy 9 can survive into young adulthood (Li et al., 2021).

We used CNV-seq to examine the placental tissue, skin tissue, umbilical cord blood, kidney, and heart of the fetus in this study. The results showed that the fetus had complete trisomy 9. The examination revealed multiple anomalies (Supplementary Figure S1). The fetus’s face showed typical indications of trisomy 9. The face was dysmorphic, with a broad forehead, blepharophimosis, low-set malformed ears with small lobes, a prominent nose with a bulbous tip, and micrognathia. The fetus’s mouth was similar to a fish’s mouth, and the fetus had a broad neck, postural anomalies, broad thumbs, and clubfeet. Both renal malformations were connected. Limited joint movement was observed.

The CNV-seq assay results of the placenta, fetal skin tissue, umbilical cord blood, kidney, and heart indicated trisomy 9. Therefore, the most likely cause of complete trisomy 9 was the non-disjunction of chromosome 9 during the diplotene phase of meiosis I. Complete trisomy 9 results from non-disjunction at meiosis and almost always occurs *de novo*. Almost all parents of fetuses with complete trisomy 9 have normal chromosomes. In addition, it cannot be ruled out that some phenotypically normal parents actually have mosaicism, with only a small percentage of abnormal cells, such as in some tissues or ovaries. Trisomy 9 cells in the ovary may lead to the birth of children with trisomy 9. Circumstantial evidence is scarce due to the normal phenotypes of the parents.

The prenatal diagnosis of trisomy 9 makes genetic counseling difficult since we must know the abnormal manifestations of various systems in fetuses with trisomy 9. To enhance our understanding, we conducted a comprehensive literature review on complete trisomy 9, including the addition of the case in our study. To date, a total of 59 cases of complete trisomy 9 have been reported. The first documented case of complete trisomy 9 was described in 1973 by Feingold and Atkins, wherein, remarkably, the male infant survived for 28 days despite presenting with multiple abnormalities (Feingold and Atkins, 1973). In 1978, one infant with trisomy 9 survived the longest (107 days) and had a karyotype of 47,XY,+9q- (Mace et al., 1978). Due to the presence of multisystem dysmorphism in their fetuses, 24 pregnant women chose to undergo induced abortion; 16 infants with complete trisomy 9 died shortly after birth, without passing through the neonatal period and 11 fetuses died *in utero*. Complete trisomy 9 has a lethal prognosis (Table 1).

**TABLE 1:** Reported cases of complete trisomy 9.

No.	References	Maternal/paternal age	Year	Outcome
1	Feingold and Atkins (1973)	24/25	1973	Survived 28 days
2	Kurnick et al. (1974)	24	1974	Survived 23 days
3	Francke et al. (1975)	40	1975	TOF at 20 weeks
4	Sutherland et al. (1976)	28	1976	Survived 17 days
5	Seabright et al. (1976)	24/24	1976	Survived 16 h
6	Mace et al. (1978)	31/51	1978	Survived 107 days
7	Annerén and Sedin (1981)	32/33	1981	Survived 58 h
8	Mantagos et al. (1981)	17/18	1981	Survived 8 h
		23/22		Survived 2 h
9	Carpenter and Tomkins (1982)	—	1982	—
10	Frohlich (1982)	45/41	1982	TOF at 19 weeks
11	Romain and Sullivan (1983)	—	1983	Survived 5 days
12	Williams et al. (1985)	31/30	1985	Survived 1.5 h
13	Delicado et al. (1985)	—	1985	—
14	Marino et al. (1989)	28/30	1989	Survived 3 days
15	Benacerraf et al. (1992)	42	1992	TOF
		27		Survived 24 days
16	Raffi et al. (1992)	—	1992	—
17	Golden and Schoene (1993)	27	1993	Survived 23 days
		32		Survived 7 days
18	Satge et al. (1994)	28	1994	TOF at 30 weeks
19	McDuffie (1994)	29	1994	IUFD
20	Chitayat et al. (1995)	30	1995	Spontaneously aborted at 14 weeks
		37		TOF at 21 weeks
		22		TOF at 19 weeks
		31		Death occurred within minutes
		36		TOF at 20 weeks
21	Lam et al. (1998)	41	1998	TOF
22	Seller et al. (1998)	28	1998	TOF at 28 weeks
23	Pinette et al. (1998)	25	1998	Spontaneously aborted at 17 weeks
24	Roshanfekr et al. (1998)	28	1998	TOF at 37 weeks
25	Sandoval et al. (1999)	28	1999	IUFD at 33 weeks
26	Murta et al. (2000)	30	2000	IUFD at 15 weeks
27	Khan et al. (2001)	30	2001	—
28	Yeo et al. (2003)	32	2003	IUFD
29	Suzumori et al. (2003)	38/57	2003	IUFD at 37 weeks
30	Stevenson et al. (2004)	40	2004	Survived 1 h
31	Khoury-Collado et al. (2004)	26	2004	TOF at 17 weeks
32	Chen et al. (2004)	25/29	2004	TOF at 22 weeks
33	Quigg et al. (2005)	39	2005	—
34	Kor-Anantakul et al. (2006)	31/35	2006	Died within a few minutes after birth
35	Lim et al. (2006)	29	2006	TOF at 22 weeks
36	Nakagawa et al. (2006)	23	2006	IUFD at 36.6 weeks
37	Ferreres et al. (2008)	25	2008	IUFD at 37 weeks
		35		TOF at 22 weeks
		39		TOF at 21.6 weeks
38	Kannan et al. (2009)	31/34	2009	Survived 20 days
39	Perez et al. (2009)	26	2009	TOF at 24 weeks
40	Zuzarte et al. (2011)	30	2011	TOF at 14 weeks
41	Tonni and Grisolia (2013)	32	2013	TOF at 22 weeks
42	Tonni et al. (2014)	37	2014	TOF at 22 weeks
		28		TOF at 23 weeks
43	Pruksanusak et al. (2014)	37	2014	TOF at 21 weeks
44	Ben Slama et al. (2016)	41	2016	TOF at 20 weeks
45	Zhang et al. (2021)	44	2021	—
46	Fuma et al. (2022)	34/31	2022	Survived 49 min
47	Li et al. (2022)	28	2022	TOF at 23 weeks
		34		IUFD at 22 weeks
48	The present case	37		TOF at 24 weeks

TOP, termination of pregnancy; IUFD, intra uterine fetal death.

As shown in Table 2, the highest proportion of pregnant women were aged 23–29 years (21 cases). Only 16 of these pregnant women were aged ≥35 years old. Among the cases reported in the literature, it is worth noting that 39 instances of trisomy 9 were documented in mothers who were younger than 35 years old, rather than in those of advanced maternal age. This observation suggests that the occurrence of trisomy 9 does not appear to be significantly correlated with maternal age.

**TABLE 2:** Maternal age distribution of fetuses with trisomy 9.

Maternal age range	Number of pregnant women
17 ≤ age ≤ 23	4
23 < age ≤ 29	21
29 < age ≤ 35	15
35 < age ≤ 41	12
41 < age ≤ 47	3
Total	55

Fetuses with complete trisomy 9 exhibit anomalies in multiple systems, as summarized in Table 3. Notably, head and face abnormalities are the most prominent features. These included malformed low-set ears (50 cases, 84.75%), micrognathia/microretrognathia (45 cases, 77.97%), a bulbous nose (27 cases, 45.76%), cleft lip/palate (25 cases, 42.37%), and microphthalmia (20 cases, 33.90%). Among the cardiovascular system malformations, ventricular septal defects (28 cases, 47.46%) were the most frequently observed. In terms of the skeletal system and limbs, absent or hypoplastic toes/phalangeal/tarsal/calcaneal bones (27 cases, 45.76%), overlapping fingers (21 cases, 35.59%), malformed hands/clenched hands (19 cases, 32.20%), and rocker-bottom feet (19 cases, 32.20%) were the most common abnormalities. Dandy-Walker malformation (14 cases, 23.73%) was the most frequent malformation in the central nervous system, while horseshoe kidney (10 cases, 16.95%) and hypoplastic kidneys (10 cases, 16.95%) were the most common malformations in the urinary system. In male and female fetuses, the most frequent malformations were short penis/small penis (13 cases, 22.03%) and bicornuate uterus (3 cases, 5.08%), respectively. Abnormal lobation (12 cases, 20.34%) was the most common malformation in the respiratory system and diaphragm. A supernumerary spleen (6 cases, 10.17%) was the most frequently observed malformation in the hematopoietic and lymphoid system. For the gastrointestinal system, intestinal malrotation (7 cases, 11.86%) and gallbladder or bile duct hypoplasia/agenesia (7 cases, 11.86%) were the most frequent malformations. Lastly, adrenal hypoplasia (7 cases, 11.86%) was the most common malformation in the endocrine system. Fifty-nine cases with malformations in various systems had malformation rates of more than 30% for malformed low set ears, micrognathia/microretrognathia, a bulbous nose, cleft lip/palate, microphthalmia, absent or hypoplastic toes/phalangeal/tarsal/calcaneal bones, clinodactyly/overlapping finger, malformed hands/clenched hands, rocker-bottom feet, and ventricular septal defect.

**TABLE 3:** Abnormal manifestations of various systems in complete trisomy 9 cases.

Head and neck	Number	Percentage	Skeletal system and limbs	Number	Percentage
Malformed low set ears	50	84.75%	Absent or hypoplastic toes/phalangeal/tarsal/calcaneal bones	27	45.76%
Micrognathia/microretrognathia	46	77.97%	Clinodactyly/overlapping fingers	21	35.59%
Bulbous nose	27	45.76%	Malformed hands/clenched hands	19	32.20%
Cleft lip/palate	25	42.37%	Rocker-bottom feet	19	32.20%
Microphthalmia	20	33.90%	Abnormal palmar creases/Simian creases	14	23.73%
Large fontanelles/wide cranial sutures	14	23.73%	Clubfeet	13	22.03%
Short webbed neck	14	23.73%	Restricted joints	11	18.64%
Microcephaly	12	20.34%	Abnormal/hypoplastic nails	10	16.95%
Hypertelorism	10	16.95%	Absent or hypoplastic pubic bones	7	11.86%
Enophtalmos	8	13.56%	Hypoplastic long bones	7	11.86%
Broad flattened nose	7	11.86%	Deformed/abnormal number of ribs (12/13)	6	10.17%
High arched palate	6	10.17%	Dislocated elbows	5	8.47%
Fetal nuchal translucency thickness	6	10.17%	Dislocated ankles	5	8.47%
Globular head/macrocrania	4	6.78%	Sacral dimple	4	6.78%
Corneal opacities	3	5.08%	Thin skull bones	4	6.78%
Small nose	2	3.39%	Hypoplasia iliac bone	3	5.08%
Saddle nose	2	3.39%	Dislocations of shoulder	3	5.08%
Choanal atresia	2	3.39%	Absent sacral segment	2	3.39%
Strawberry head	2	3.39%	Bulbous fingertips	2	3.39%
Triangular head	2	3.39%	Feet foreshortened	2	3.39%
Unilateral atresia of auditory meatus	2	3.39%	Hyperextensible joints	2	3.39%
A high forehead	2	3.39%	Muscular hypotonia	2	3.39%
Iris coloboma	2	3.39%	Narrow thorax	2	3.39%
Absent tongue	2	3.39%	Hyperconvex nails	1	1.69%
Hypoglossia	2	3.39%	Talipes calcaneovalgus	1	1.69%
Gingival hyperplasia	2	3.39%	Dislocation radius	1	1.69%
Low hairline	2	3.39%	Dislocated knees	1	1.69%
Narrowing forehead	1	1.69%			
Frontal bossing	1	1.69%			
Flat frontal bone	1	1.69%			
Broad forehead	1	1.69%			
Upslanting palpebral fissures	1	1.69%			
Plagiocephaly	1	1.69%			
Upward-slanted eyes	1	1.69%			
Bitemporal narrowing	1	1.69%			
Receding hairline	1	1.69%			
Pinpoint pupils	1	1.69%			
Mouth large and downturned at the corners	1	1.69%			
Microstomia	1	1.69%			
Scalp edema	1	1.69%			

Cardiovascular system	Number	Percentage	Central nervous system	Number	Percentage
Ventricular septal defect	28	47.46%	Dandy-Walker malformation	14	23.73%
Atrial septal defect	8	13.56%	Ventriculomegaly/hydrocephaly	10	16.95%
Patent ductus arteriosus	7	11.86%	Agenesis or hypoplasia of the corpus callosum	9	15.25%
Single umbilical artery	8	13.56%	Cerebellar vermian defect/hypoplastic	5	8.47%
Valve dysplasia	7	11.86%	Neural tube defects	5	8.47%
Truncus arteriosus	4	6.78%	Cystic spina bifida	3	5.08%
Persistent left superior cava	4	6.78%	Pachygyria/anomalous sulcation	2	3.39%
Hypoplastic left ventricle and ascending aorta	4	6.78%	Syryngomyelia	2	3.39%
Ventricular hypertrophy	4	6.78%	Abnormal neuronal migration	2	3.39%
Double outlet right ventricle	3	5.08%	Acral vertebral anomalies	1	1.69%
Aortic stenosis	2	3.39%	Bifid choroid plexus	1	1.69%
Endocardial fibroelastosis	2	3.39%	Cyst in midline of tentorium	1	1.69%
Enlarged heart	2	3.39%	Widely separated cerebellar hemispheres, large dilated fourth ventricle	1	1.69%
Patent foramen ovale	2	3.39%	Cephalic ventricular dilatation	1	1.69%
Dilation the pulmonary valve/aortic	2	3.39%			
Valvular pulmonary stenosis	1	1.69%			
Right ventricular chamber was dilated	1	1.69%			
Abnormal venous drainage	1	1.69%			
Dextrocardia	1	1.69%			
Varix umbilical vein	1	1.69%			
Pericardial effusion	1	1.69%			
Dilated coronary sinus	1	1.69%			
Overriding aorta	1	1.69%			
Abnormal cardiac calcification	1	1.69%			

Urinary system	Number	Percentage	Genital anomalies in male fetus	Number	Percentage
Horseshoe kidney	10	16.95%	Short penis/small penis	13	22.03%
Hypoplastic kidneys	10	16.95%	Cryptorchidism	10	16.95%
Hydronephrosis	7	11.86%	Hypoplastic scrotum	9	15.25%
Microscopic cysts in renal cortex	3	5.08%	Hypospadias	3	5.08%
Renal ectopia	2	3.39%	Histologically abnormal testes	1	1.69%
Mega-ureter	2	3.39%	Genital anomalies in female fetus	Number	Percentage
Dilatation of the glomeruli and tubules	1	1.69%	Bicornuate uterus	3	5.08%
Absent/non-functional renal arteries	1	1.69%	Female hypoplastic genitalia, short distance between anus and vulva	1	1.69%
Severe renal failure	1	1.69%	Elongated ovaries	1	1.69%
Bilateral pyelectasis	1	1.69%			
Hypoplastic bladder	1	1.69%			

Respiratory system and diaphragm	Number	Percentage	Hematopoietic and lymphoid system	Number	Percentage
Abnormal lobation	12	20.34%	Supernumerary spleen	6	10.17%
Pulmonary hypoplasia	7	11.86%	Thymic hypoplasia/aplasia	4	6.78%
Focal bilateral acute bronchopneumonia/pneumonia	3	5.08%	Dilatation of subpleural and interlobular lymphatics	1	1.69%
Interlobular septal edema	1	1.69%			

Gastrointestinal system	Number	Percentage	Endocrine system	Number	Percentage
Intestinal malrotation	7	11.86%	Adrenal hypoplasia	7	11.86%
Gallbladder or bile ducts hypoplasia/agenesia	7	11.86%	Enlarged thyroid, adrenal cytomegaly	1	1.69%
Imperforate anus	5	8.47%	Thymic hypoplasia	1	1.69%
Umbilical hernia	5	8.47%			
Liver calcifications	4	6.78%			
Agenesis of dorsal pancreas	3	5.08%			
Annular pancreas	2	3.39%			
Cholestasis	2	3.39%			
Fatty changes of the liver	2	3.39%			

Severe fetal malformations are typically identified during the second or third trimester of pregnancy or after birth. The head and neck regions are the most commonly affected areas by these anomalies (Table 3). Although these studies show the rate of malformation in each system, many of them did not perform further autopsy or did not observe the phenotypes in each system. It is possible that statistically significant abnormal phenotypes are easier to observe. There were six cases of thickened NT. Previous ultrasound technology was not advanced; therefore, in many cases, NT was not measured rather than NT not being thickened. In addition, many studies were not described in detail. For example, some studies mentioned cardiac abnormalities, among which only congenital heart disease was mentioned without being described in detail (Benacerraf et al., 1992; Seller et al., 1998; Zhang et al., 2021).

Based on the common malformations associated with trisomy 9 syndrome mentioned earlier, targeted sonographic examinations can be conducted to detect multiple abnormalities. These include microcephaly, Dandy-Walker malformation, abnormal facial features (such as malformed low-set ears, a hypoplastic nose, micrognathia, and cleft lip/palate), congenital heart defects (such as ventricular septal defect, atrial septal defect, cardiomegaly, double outlet right ventricle, and valvular pulmonary stenosis), abnormal limbs (such as malformed hands and clubfeet), and a single umbilical artery.

It is important to note that a morphological description alone cannot replace the diagnosis of complete trisomy 9 through karyotype analysis. However, if some of the characteristic features of trisomy 9 mentioned here are observed, this syndrome should be suspected. While 59 cases of complete trisomy 9 have been reported in the literature, it is encouraged to continue reporting new findings to further enhance our understanding of the morphological characteristics across various systems in fetuses with this syndrome.

## Data Availability

The original contributions presented in the study are included in the article/Supplementary Material, further inquiries can be directed to the corresponding author.
