# Supplementary figures and images for: Case report: A case report and literature review of complete trisomy 9
Source: Front Genet. 2023 Aug 31;14:1241245. doi: 10.3389/fgene.2023.1241245 (PMC10500842; doi:10.3389/fgene.2023.1241245)

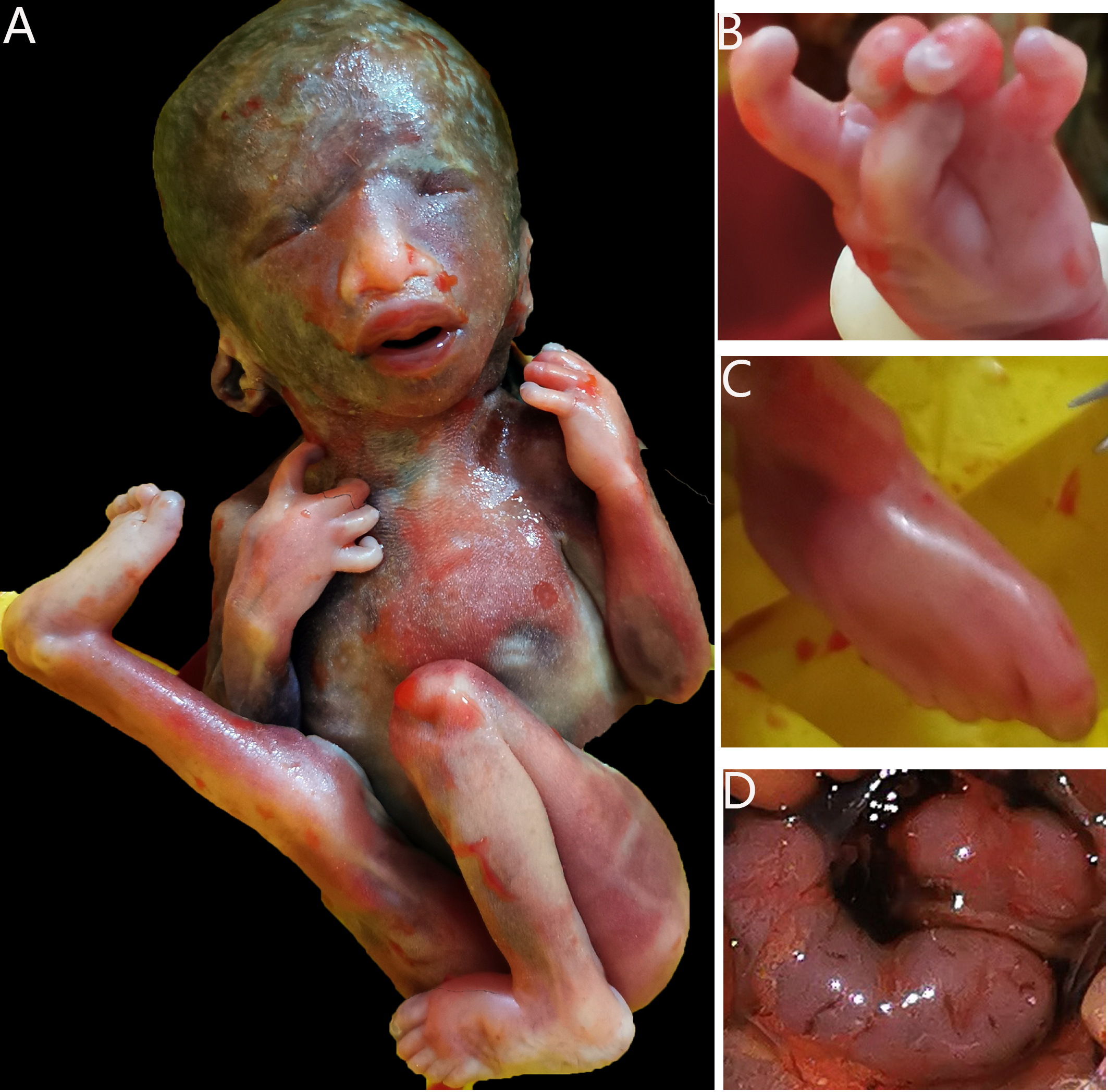

Supplement: Supplementary file 1 [file Image1.TIF]
